# Molecular Detection and Characterisation of *Coxiella burnetii* in Koala (*Phascolarctos cinereus*) Urogenital Tract Swabs †

**DOI:** 10.3390/pathogens13100873

**Published:** 2024-10-04

**Authors:** Karen O. Mathews, David Phalen, Paul A. Sheehy, Jacqueline M. Norris, Damien P. Higgins, Katrina L. Bosward

**Affiliations:** 1 Sydney School of Veterinary Science, The University of Sydney, Sydney, NSW 2006, Australia; karen.mathews@sydney.edu.au (K.O.M.); david.phalen@sydney.edu.au (D.P.); paul.sheehy@sydney.edu.au (P.A.S.); jacqui.norris@sydney.edu.au (J.M.N.); damien.higgins@sydney.edu.au (D.P.H.); 2 Koala Health Hub, Sydney School of Veterinary Science, The University of Sydney, Sydney, NSW 2006, Australia; 3 Sydney Infectious Diseases Institute, The University of Sydney, Sydney, NSW 2006, Australia

**Keywords:** Australia, *Coxiella burnetii*, koalas, Q fever, urogenital swabs, wildlife rehabilitators

## Abstract

Q fever is a zoonosis caused by *Coxiella burnetii*, primarily affecting those in close contact with domestic ruminants, the main source of human infection. *Coxiella burnetii* has also been detected in various wildlife species globally. In Australia, serological and molecular studies have shown exposure to and infection by *C. burnetii* in macropods, bandicoots, and koalas. However, the extent to which these species contribute to human infection remains unclear. An unpublished public health investigation into a Q fever case in a person involved in koala care could not conclusively link the infection to koalas due to the patient’s broad animal exposure. This study aimed to explore the potential role of koalas in transmitting *C. burnetii* to humans by investigating the presence of *C. burnetii* DNA in urogenital tract (UGT) swabs from koalas. DNA was extracted from UGT swabs from koalas in three regions in New South Wales, Australia. An optimised multiplex qPCR assay detected *C. burnetii* DNA in 2 out of 225 samples (0.89%) at approximately 10 genome equivalents per reaction. Both positive samples amplified all three gene targets. MLVA genotyping identified two distinct *C. burnetii* genotypes previously isolated from Australian Q fever cases. These findings highlight the need for vaccination against Q fever for those in close contact with koalas.

## 1. Introduction

Q fever is a zoonotic disease caused by *Coxiella burnetii*, an obligate intracellular pathogen from the gamma subdivision of Proteobacteria in the *Coxiellaceae* family [1]. The disease was initially described in abattoir workers in Queensland, Australia, in 1935 [2], but has since been reported throughout the world, except for New Zealand [3]. In the environment *C. burnetii* exists as a highly infectious extracellular spore-like form that persists in the environment for up to 150 days [4,5,6] and can easily be dispersed by the wind over long distances [7]. Infection is mostly acquired following inhalation of *C. burnetii* contaminated aerosols or dust [4,8,9].

In humans, the clinical manifestations of *C. burnetii* infection are broad, ranging from asymptomatic seroconversion in 20–80% of cases depending on geographical region to acute disease, which typically presents as a self-limiting ‘influenza-like’ illness, characterised by high fevers, headaches, chills, and fatigue, with hepatitis and pneumonia as potential complications [10]. Post-Q fever fatigue syndrome and persistent focal infection (previously ‘chronic Q fever’) are well recognised sequelae of *C. burnetii* infection, which may manifest years after primary infection, regardless of the initial clinical presentation [9,11]. In Australia, Q fever has been nationally notifiable since 1977 [12], with approximately 500 human cases reported annually [13]. Australia is the only country where an effective licensed human Q fever vaccine (Q-Vax^®^; Seqirus, Parkville, VIC, Australia) is available and vaccination is recommended for those engaged in high-risk occupations, including abattoir workers, veterinarians, and zoo and wildlife workers [14].

Domestic ruminants are considered the major reservoirs for human infection [15]. Infected ruminants contaminate the environment by shedding *C. burnetii* in their milk, urine, faeces, and, to a greater extent, products of conception [5]. Serological and molecular studies have demonstrated both *C. burnetii* exposure and infection in many wildlife species globally [16] and specifically in Australian wildlife species including bandicoots, possums, flying foxes [17,18], and macropods [19,20,21,22]. As such, it has been suggested that wildlife may also be a potential source of *C. burnetii* infection for humans. To date, there have been no *C. burnetii* serological studies in koalas (*Phascolarctos cinereus*). In the only published molecular study of *C. burnetii* in koalas, 99 koala samples were tested in two singleplex qPCR assays targeting *com1* (the outer membrane protein-coding gene) and the multicopy insertion sequence gene IS*1111*. *Coxiella burnetii* DNA was detected in 3/26 (11.5%) blood, 1/43 (2.33%) faecal, and 1/30 (3.3%) urine samples, corresponding to an overall *C. burnetii* DNA detection rate of 5.1% [18]. These findings raise the question as to whether people in close contact with koalas are potentially at risk of contracting Q fever, particularly those people caring for koalas with chlamydiosis caused by *Chlamydia pecorum*, a significant urinary tract pathogen in koalas [23]. People diagnosing, treating, or rehabilitating koalas with chlamydiosis may have direct contact with urogenital tract secretions, excretions, and their associated aerosols, which could also potentially be contaminated with *C. burnetii*. To date, there have been no published cases of Q fever in humans where the source of infection was conclusively identified as koalas. An Australian unpublished public health investigation into a case of medically diagnosed Q fever in a person extensively involved in the care and rehabilitation of koalas was unable to conclusively rule koalas in or out as the source of *C. burnetii* exposure, largely due to the patient’s broad range of animal and environmental exposures and the delay associated with the Q fever incubation period, which is known to be up to 32 days [24].

Demonstration of the presence of *C. burnetii* DNA in koala samples is the first step towards determining whether koalas can become infected with, and subsequently shed, *C. burnetii* in their secretions and excretions, and to furthering our understanding of their role as a source of infection for humans. Therefore, this study aimed to investigate for the presence of *C. burnetii* DNA in UGT swabs obtained from koala populations in NSW, Australia, using an optimised multiplex qPCR assay. Urogenital tract swabs were chosen as a sample to assess shedding via the koala reproductive tract but also because they are routinely used to diagnose chlamydiosis in koalas and, therefore, represent a feasible potential exposure route for humans working with koalas.

## 2. Materials and Methods

### 2.1. Sample Source and Sample Size Calculation

**Figure 1:**
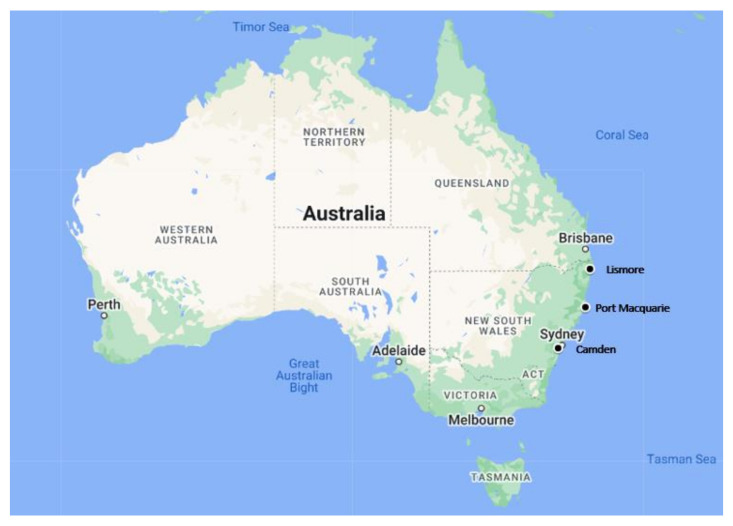
Geographical location, in New South Wales, Australia, from which urogenital tract swabs of koalas (*Phascolarctos cinereus*) were obtained to investigate for the presence of *Coxiella burnetii* DNA. The study locations (Lismore, Port Macquarie, and Camden) are indicated by solid dots on the above map.

This study utilised archived surplus DNA previously extracted from urogenital tract (UGT) swabs, collected from the urethra or urogenital sinus of male and female koalas, respectively. These swabs were submitted to The Koala Health Hub (KHH) in the Sydney School of Veterinary Science at the University of Sydney between 2016 and 2020 for chlamydial diagnostic testing for clinical management purposes. Swabs were collected from koalas admitted to three wildlife rehabilitation facilities in NSW, Australia (Lismore, Port Macquarie, and Camden; Figure 1). Ethics approval was not required as the de-identified samples were collected and submitted for diagnostic and clinical management purposes, independent of the researchers. Sample size determination was based on a *C. burnetii* DNA prevalence of 5%, as determined by the only previous study of *C. burnetii* in koalas [18]. Assuming 5% *C. burnetii* DNA detection, a total of 225 samples (74–76 randomly selected from each location) were required for estimating *C. burnetii* DNA prevalence in koalas with 5% precision and 95% confidence [25].

### 2.2. DNA Extraction and PCR

Genomic DNA was extracted from koala UGT swabs, as part of a prior research project, using a MagMAX™CORE Nucleic Acid Purification Kit (Thermo Fisher Scientific North Ryde, NSW, Australia) and a robotic workstation (KingFisher Flex, Thermo Fisher Scientific, North Ryde, NSW, Australia). The tips of the swabs were removed using clean bleached sterile scissors and placed into a microcentrifuge tube containing 350 µL of MagMAX CORE Lysis Solution and 10 µL of Proteinase K, after which they were briefly vortexed and incubated at 56 °C for at least 60 min. Following transfer of the lysate to a 96-deepwell plate, the samples were processed according to the manufacturer’s instructions. DNA was eluted in 100 µL of elution buffer and extraction controls (ECs) were included in every extraction run.

### 2.3. Molecular Detection of Host Species and Coxiella burnetii DNA

#### 2.3.1. Detection of Koala DNA (Endogenous Control)

The detection and quantification of koala DNA in extracted samples was performed using a singleplex qPCR assay targeting the *Phascolarctos cinereus* (koala) β-actin gene [26] (Koala β-actin). Each reaction contained 10 µL 1X SensiFAST Probe No-ROX Kit (BioLine, Alexandria, NSW, Australia), primers and probe (synthesised by Macrogen Inc., Seoul, Republic of Korea; Table 1), 2 µL DNA, and nuclease-free water in a total volume of 20 µL. Amplification and fluorescence detection was performed in a Bio-Rad-CFX Real-Time PCR Thermocycler (Bio-Rad laboratories Pty Ltd., Gladesville, NSW, Australia) according to the following cycling parameters: initial denaturation at 95 °C for 3 min, followed by 40 cycles of denaturation at 95 °C for 10 s, and at 58 °C for 40 s. Positive controls and no-template controls (NTCs) with water in place of DNA were included in each PCR run. Samples were classified as positive if amplification occurred at a quantification cycle (Cq) < 32. Each sample was screened for the presence of inhibitors by performing PCR on a neat and 1/10 dilution. Samples demonstrating the presence of inhibitors (identified when the difference between the Cq of the neat and the 1/10 dilution was <3.0 cycles) were diluted 1/10 before testing for the presence of *C. burnetii* DNA.

#### 2.3.2. Detection of *Coxiella burnetii* DNA in Koala Urogenital Swabs

A multiplex qPCR (CoxMP) developed using the Minimum Information for Publication of Quantitative Real-Time PCR Experiments (MIQE) guidelines [30] (Bustin et al., 2009) was used to test for the presence of *C. burnetii* DNA in the koala UGT swab DNA extracts. The CoxMP assay containing two single-copy genes, *groEL* (heat shock operon; *htpAB*) and *com1* (the outer membrane protein-coding gene), and the multicopy insertion sequence gene IS*1111* (Table 1), was optimised and validated using commercially available *C. burnetii* control DNA (Nine Mile RSA493; Amplirun^®^ Vircell, Granada, Spain). The lower limit of detection for these qPCR assays was determined to be 11 copies of the *C. burnetii* genome per reaction, which corresponded to a Cq of ~34, ~36, and ~35 for IS*1111*, *com1*, and *htpAB*, respectively. Each reaction contained 5 µL 1X SensiFAST Probe No-ROX Kit (BioLine, Alexandria, NSW, Australia), primers and probe (synthesised by Integrated DNA Technologies, Baulkham Hills, NSW, Australia), 2 µL DNA, and nuclease-free water in a total volume of 10 µL. Amplification and fluorescence detection were performed as described for Koala β-actin, with annealing and extension at 60 °C. Each qPCR run included NTC and positive controls containing 1, 100, 110, and 11 copies of the *C. burnetii* genome per reaction (Amplirun^®^ Vircell, Granada, Spain). Samples were initially screened as a single qPCR reaction, and any sample producing amplification for any gene target was repeated in triplicate.

#### 2.3.3. Sample Classification Criteria

Samples were classified as positive for *C. burnetii* DNA if they were amplified reproducibly in triplicate reactions for all three gene targets and produced Cqs at or below the pre-determined Cq cut-off (representing 11 *C. burnetii* genome copies) for each assay. Samples were classified as suspect for *C. burnetii* DNA if they amplified reproducibly in triplicate reactions for all three gene targets and produced Cqs above the pre-determined Cq cut-off for each assay, or amplification across triplicates within each assay was not reproducible despite it being present for all three gene targets with Cqs at or below the pre-determined Cq cut-off for each assay. Samples were classified as negative for *C. burnetii* DNA if amplification was not observed for any gene target in the single or triplicate PCR reactions. Samples that reproducibly amplified only one gene target in the triplicate reactions were also classified as negative irrespective of the Cq.

#### 2.3.4. Sample Characterisation via Multi-Locus Variable Number of Tandem Repeat Analysis

Multi-locus variable number of tandem repeat analysis (MLVA) was undertaken to characterise samples classified as positive or suspect for the presence of *C. burnetii* DNA using three MLVA loci (ms24, ms28, and ms33) which have been demonstrated as suitable for discriminating between Australian *C. burnetii* isolates [26]. Each reaction contained 4 µL 1X MyTaq HS DNA Polymerase reaction buffer, 1U MyTaq HS DNA Polymerase (BioLine, Alexandria, NSW, Australia), 400 nM primers with the 5′ end of the forward primer from each locus labelled with FAM 6-Carboxyfluorescein (synthesised by Integrated DNA Technologies, Baulkham Hills, NSW, Australia; Table 2), 4 µL DNA, and nuclease-free water in a total volume of 20 µL. Amplification was performed using conventional PCR in a Bio-Rad T100 Thermal Cycler PCR and a96-well block (Bio-Rad laboratories Pty Ltd., Gladesville, NSW, Australia), according to the following cycling parameters: initial denaturation at 95 °C for 3 min, followed by 40 cycles of 15 s denaturation, annealing and extension at 95 °C, 58 °C, and 72 °C, respectively, and a final extension 72 °C for 10 min. All genotyping runs included a positive control (*Coxiella burnetii* Nine Mile Clone 4 DNA; NMC4) and a NTC in which water was used in place of DNA. PCR amplicons were visualised on a 2% agarose gel (VWR Life Science, Radnor, PA, USA) containing RedSafe™ nucleic acid stain (Sigma Aldrich, Castle Hill, NSW, Australia), and their approximate size was determined using a 100 bp molecular weight marker (Bioline, Alexandria, NSW, Australia). Amplicons ranging from 150 bp to 300 bp were sent to the Australian Genomic Research Facility (Victorian Comprehensive Cancer Centre, Melbourne, VIC, Australia) for sizing via capillary electrophoresis. The number of repeats for each locus was determined by comparing the amplicon size with the control strain, NMC4, which has a known repeat profile of 27-6-9 for the ms24, ms28, and ms33 loci, respectively (http://mlva.i2bc.paris-saclay.fr/MLVAnet/spip.php?rubrique50, accessed on 20 March 2024).

## 3. Results

A total of 225 koala UGT swabs collected from 225 individual animals (sampled once) were analysed in this study. The breakdown of the number of animals and sex for each geographical location is provided in Table 3.

### 3.1. Quantitative PCR Detection of Koala β-Actin (Endogenous Control)

All 225 koala UGT swab DNA extracts produced positive amplification for Koala β-actin, verifying the presence and integrity of the DNA. No inhibition was observed by comparison of the neat and 1/10 dilutions. All ECs and NTCs were determined to be negative for Koala β-actin DNA.

### 3.2. Quantitative PCR Detection of Coxiella burnetii DNA

When assayed in the CoxMP in triplicate, one (1/225; 0.44%) koala from Port Macquarie was classified as positive, and one koala (1/225; 0.44%), also from Port Macquarie, was classified as suspect (Table 4). The sex of these two animals was unavailable. The remaining 221 (221/225; 94.0%) samples from 221 animals did not amplify any gene target in the CoxMP qPCR and were classified as negative for the presence of *C. burnetii* DNA. All ECs and NTCs were determined to be negative in the CoxMP qPCR.

### 3.3. Multi-Locus Variable Number of Tandem Repeat Genotype Analysis

Results of the MLVA genotyping analysis of the positive and suspect samples are presented in Table 5. The NMC4 control strain returned an expected repeat profile of 27-6-9 for the ms24, ms28, and ms33 loci, respectively, validating the genotyping methodology. All three MLVA loci were amplified from the positive and suspect UGT DNA extracts, the repeat profiles of which revealed two different genotypes.

## 4. Discussion

This study used an optimised multiplex qPCR assay to investigatefor the presence of *C. burnetii* DNA in koala UGT swabs collected from koala populations in three geographical locations in NSW, Australia. *Coxiella burnetii* DNA was detected in DNA extracts obtained from two koalas from Port Macquarie. Additionally, MLVA genotyping identified two distinct *C. burnetii* genotypes that have been isolated from Australian clinical Q fever cases.

Rehabilitating koalas was not identified as a risk factor for *C. burnetii* exposure in previous epidemiological investigations [32,33] and koalas are not typically reported in association with Q fever notifications. However, *C. burnetii* DNA detection in koala UGT swabs in this study suggests that koalas may represent a potential source of *C. burnetii* for humans. The only other study to investigate *C. burnetii* infection in koalas was conducted in a different geographical location (Queensland versus NSW for the current study) reported detecting *C. burnetii* DNA in the blood, faeces, and urine of koalas [18]. The DNA detection rate of 5% reported by Tozer et al. [18] was approximately five times higher than the 0.8% found in the current study. However, comparing the current findings to those reported by Tozer et al. [18] is difficult due to the limitations associated with PCR methodology and the lack of information regarding Cqs and cut-offs provided. Future studies investigating for the presence of *C. burnetii* DNA should consider using multiplex qPCR assays (such as the CoxMP) with stringent pre-determined cut-offs and classification criteria using multiple gene targets to enhance sensitivity. Furthermore, the detection of *C. burnetii* DNA and the classification of samples as positive based on the amplification of IS*1111* alone is not recommended as this gene target has been found in Coxiella-like endosymbionts [34,35,36]. Therefore, studies employing this strategy may report false-positive *C. burnetii* DNA detection. Standardisation of molecular techniques will also enable the comparison of results across research groups. Genotyping of positive samples is also recommended to further characterise *C. burnetii* strains and their relevance to public health.

The two koala UGT samples classified as positive and suspect for the presence of *C. burnetii* DNA amplified at concentrations of approximately 11 genome equivalents (GEs) per reaction. Assuming ≤10 genome equivalents per reaction and 100% yield from DNA extraction, this extrapolates to ≤1000 organisms per swab. Additionally, the presence of bacterial DNA by PCR does not guarantee viability. Nevertheless, given the low dose of approximately 10–15 organisms required to infect humans [37], secretions and excretions from the UGT and gastrointestinal tracts of koalas should still be considered to represent a plausible risk for *C. burnetii* exposure for those in close contact with these animals and involved in activities such as cage cleaning or collecting UGT swabs for routine diagnostics (e.g., diagnosis of chlamydia or other infections). Further to these considerations, MLVA genotyping on the positive and suspect koala samples identified two different genotypes of *C. burnetii* that have been previously identified from Australian human clinical Q fever cases in New South Wales [38], further demonstrating the public health relevance of these koala *C. burnetii* strains. Taken together, all these considerations reinforce the need for those working closely with Australian wildlife to be vaccinated against Q fever as per national guidelines [14] and as recommended in previous studies [32,33].

Urogenital swabs may represent a useful sampling technique to identify *C. burnetii*-infected koalas. Given *C. burnetii* is an intracellular pathogen, a double swabbing technique should be utilised which enables a greater number of epithelial cells on the second swab after the removal of the surface exudate by the first [39]. The finding of *C. burnetii* DNA detection in UGT swabs could indicate that shedding may be occurring via the reproductive and/or urinary tract or, potentially, contamination from the intestinal tract. Future studies could be directed to establish how *C. burnetii* may potentially infect these body systems as an aid to further understanding pathogenesis in these species. Finally, future research should be directed towards obtaining samples from healthy koala populations, however, disruption to species would need to be carefully considered.

## 5. Conclusions

This is the first study to investigatefor the presence of *C. burnetii* in koala populations in New South Wales. *Coxiella burnetii* was detected at a low prevalence of <1% in the koala populations tested and at relatively low copy numbers. However, given the low infectious dose of *C. burnetii*, the consequences for those who present with the incapacitating Q fever fatigue syndrome or complications such as endocarditis that may result in death, and the finding that MLVA genotyping identified *C. burnetii* genotypes previously isolated from Australian clinical Q fever cases, it is recommended that people in close contact with koalas be vaccinated against Q fever as per Australian national guidelines [14].

## Figures and Tables

**Table 1:** Primer and probe sequences of qPCR assays used to test for the presence of koala (*Phascolarctos cinereus*) and *Coxiella burnetii* DNA in urogenital swabs obtained from koalas in New South Wales, Australia.

Species	Gene Target	Primer/Probe	Sequence (5′-3′)	Final Concentration (nM)	Amplicon Size (bp)	Reference or Accession Number
Koala (*Phascolarctos cinereus*) endogenous control	β-actin	Koalaβ-actin-F	CTCAGATTATGTTTGAGACCTTC	400	144	[26]
		Koalaβ-actin-R	CCTTCATAGATGGGCACA	400		
		Koalaβ-actin-P	^a^ FAM-ACCATCACCAGAGTCCATCACAAT-BHQ1 ^b^	200		
*Coxiella burnetii*	IS*1111* ^†^	IS*1111*-F	CGCAGCACGTCAAACCG	300	146	[27]
		IS*1111*-R	TATCTTTAACAGCGCTTGAACGTC	300		
		IS*1111*-P	^a^ FAM-ATGTCAAAAGTAACAAGAATGATCGTAAC-BHQ1 ^b^	200		
	*com1* ^‡^	*com1*-F	AAAACCTCCGCGTTGTCTTCA	400	76	[28]
		*com1*-R	GCTAATGATACTTTGGCAGCGTATTG	300		
		*com1*-P	^c^ Cy5-AGAACTGCCCATTTTTGGCGGCCA-BHQ2 ^d^	200		
	*htpAB* ^§^	*htpAB*-F	GTGGCTTCGCGTACATCAGA	300	114	
		*htpAB*-R	CATGGGGTTCATTCCAGCA	300		[29]
		*htpAB*-P	^e^ HEX-AGCCAGTACGGTCGCTGTTGTGGT-BHQ1 ^b^	200		

^a^ 6-Carboxyfluorescein, ^b^ Black Hole Quencher-1, ^c^ Cyanine Dye 5, ^d^ Black Hole Quencher-2, ^e^ HEX™ Dye Phosphoramidite, ^†^ insertion sequence *1111* (IS*1111*), ^‡^ outer membrane protein (*com1*), koala (*Phascolarctos cinereus*), ^§^ heat shock operon (*htpAB*).

**Table 2:** Primer sequences for multi-locus variable number of tandem repeat analysis (MLVA) used to characterise *Coxiella burnetii* DNA in urogenital swabs obtained from koalas (*Phascolarctos cinereus*) in New South Wales, Australia.

Loci	Primer	Sequence (5′-3′)	Final Concentration (nM)	Amplicon Size (bp)	Reference
ms24	ms24-F	^a^ FAM-ATGAAGAAAGGATGGAGGGACT	400	150–300	
	ms24-R	GCCACACAACTCTGTTTTCAG			
ms28	ms28-F	^a^ FAM-TAGCAAAGAAATGTGAGGATCG			[31]
	ms28-R	ATTGAGCGAGAGAATCCGAATA			
ms33	ms33-F	^a^ FAM-TAGGCAGAGGACAGAGGACAGT			
	ms33-R	ATGGATTTAGCCAGCGATAAAA			

^a^ 6-Carboxyfluorescein.

**Table 3:** Details of koala (*Phascolarctos cinereus*) urogenital swabs collected from individual animals and tested for the presence of *Coxiella burnetii* DNA using qPCR in New South Wales, Australia. The sex was not available for all animals as these details are not routinely supplied to the Koala Health Hub.

Geographical Location	Number of Animals		Sex					
			Female		Male		Unknown	
	n	%	n	%	n	%	n	%
Camden	75	33.3	30	47.6	33	52.4	12	16
Lismore	74	32.9	21	35	39	65	14	18.9
Port Macquarie	76	33.8	-	-	-	-	76	100

**Table 4:** Results of multiplex qPCR assay analysis in urogenital swabs obtained from two koalas (*Phascolarctos cinereus*) from Port Macquarie, New South Wales, Australia. The lower limit of detection was determined to be 11 copies of the *C. burnetii* genome per reaction, corresponding to a Cq of ~34, ~36, and ~35 for IS*1111*, *com1*, and *htpAB*, respectively.

		qPCR Assay Cqs and Cut-Offs					
Animal ID	Sex	Endogenous Control (Koala β-Actin)	*Coxiella burnetii* Multiplex	Target Gene			Sample Classification
				IS*1111* ^†^ ≤ 34	*com1* ^‡^ ≤ 36	*htpAB* ^§^ ≤ 35	
3772-8	male	21.6	Singles	30.1	34.1	32.5	positive
			Triplicates	29.7	34.1	32.8	
				29.8	33.8	33.5	
				30.0	34.4	33.1	
18-10145	unknown	29.4	Singles	33.4	36.2	34.9	suspect
			Triplicates	34	38.3	-	
				34	-	37	
				33.4	-	-	

ID: identification, qPCR: quantitative PCR, Cq: quantification cycle, ^†^ IS*1111*: insertion sequence *1111*, ^‡^ *com1*: outer membrane protein, ^§^ *htpAB*: heat shock operon.

**Table 5:** Results of multi-locus variable number of tandem repeat analysis (MLVA) genotyping of *Coxiella burnetii* DNA detected in urogenital swabs obtained from two koalas (*Phascolarctos cinereus*) from Port Macquarie, New South Wales, Australia.

Sample	MLVA Locus			Genotype
	ms24	ms28	ms33	
**Nine Mile Reference Strain**	27	6	9	Nine Mile Reference Strain
3772-8	17	5	5	CbAU06
18-10145	14	5	5	CbAU02

MLVA—multi-locus variable number of tandem repeat analysis.

## Data Availability

Data from this study are available on reasonable request.
